# Without insight accompanied with deteriorated brain functional alterations in healthy individuals with auditory verbal hallucinations: a pilot study

**DOI:** 10.1007/s11682-019-00207-3

**Published:** 2019-12-13

**Authors:** Chuanjun Zhuo, Feng Ji, Xiaodong Lin, Hongjun Tian, Lina Wang, Sha Liu, Hong Sang, Wenqiang Wang, Chunmian Chen

**Affiliations:** 1 School of Mental Health, Jining Medical University, Jining, 272119 Shandong Province China; 2 Psychiatric-Neuroimaging-Genetics Laboratory, Wenzhou Seventh People’s Hospital, Wenzhou, 325000 Zhejiang Province China; 3 Psychiatric-Neuroimaging-Genetics-Comorbidity Laboratory, Tianjin Mental Health Centre, Tianjin Anding Hospital, Mental Health Teaching Hospital of Tianjin Medical University, Tianjin, 300222 China; 4 Department of Psychiatry, First Hospital/First Clinical Medical College of Shanxi Medical University, Taiyuan, China; 5 MDT Center for Cognitive Impairment and Sleep Disorders, First Hospital of Shanxi Medical University, Taiyuan, 030001 China; 6 Department of Psychiatry, Changchun Sixth Hospital, Changchun, 130052 Jilin Province China; 7 Co-collaboration Laboratory of China and Canada, Xiamen Xianyue Hospital and University of Alberta, Xiamen, 361000 China

**Keywords:** Auditory verbal hallucinations, Healthy individuals, Insight, Global functional connectivity density

## Abstract

Few studies have reported on brain functional differences between healthy individuals with auditory verbal hallucinations (Hi-AVH) with and without insight, so we designed a study to address this knowledge gap. We enrolled 12 Hi-AVH with insight, 15 Hi-AVH without insight, and 15 AVH-free controls (Healthy controls). Global functional connectivity density (gFCD) mapping was used to estimate brain networks. We found that the most common alterations in both Hi-AVH groups were increased gFCD in superior parietal lobule and superior temporal gyrus. We also found that distinct brain functional patterns of Hi-AVH without insight comprised lower gFCD in the frontal lobe oculomotor area, dorsolateral prefrontal cortex, supramarginal gyrus, primary auditory cortex, sensorimotor cortex, ventral anterior, and posterior cingulate Our pilot findings support the hypothesis that abnormal reciprocal action in the circuits for processing perception, memory, language, and attentional control may be pathological features of auditory verbal hallucinations.

## Introduction

According to the strictest criteria proposed by Johns (“Did you at any time hear voices saying quite a few words or sentences when there was no one around that might account for it?”), 0.7% of the general population have experienced auditory verbal hallucinations (AVH) (Beavan et al. 2011; Johns et al. 2004). Of those who have experienced AVH, ones who “did not have clinically defined delusions, disorganization, or negative or catatonic symptoms, nor did they meet criteria for cluster A personality disorder” (Sommer et al. 2010) can be defined as healthy individuals with AVH (Hi-AVH). Within this population, AVH is a risk factor for psychosis or other mental disorders requiring medical care (Daalman et al. 2016).

In cases of psychosis, insight, or awareness of the disorder and its consequences, is often a strong predictor of prognosis. Previous studies of AVH in patients with schizophrenia found that lack of insight is associated with AVH deterioration and treatment difficulties. On the other hand, good insight may help alleviate the symptoms of AVH in patients with schizophrenia (Chang et al. 2018; Xavier et al. 2018; Waters 2012; Pijnenborg et al. 2013; Emami et al. 2016). Studies have reported that insight is associated with structural and functional brain alterations and that these brain alterations are in turn associated with treatment outcomes (Buchy et al. 2015; Sapara et al. 2007; Buchy et al. 2011; Bose et al. 2014). Together these studies suggest that insight and AVH are associated with distinct patterns of brain structure and function, and point specifically to regions in the temporal, frontal, and parietal lobules.

Given that Hi-AVH is a risk factor for psychosis and other mental disorders requiring medical intervention (Sommer et al. 2010; Daalman et al. 2016), investigation into the functional brain patterns involved may provide critical information to better understand the pathological features of AVH and the role of insight in disease progression. However, it is important to avoid confounding factors such as therapeutic agents and other psychotic symptoms in order to determine the precise brain patterns associated with AVH. A voxel-wise, data-driven method known as global functional connectivity density (gFCD) mapping is widely used to test the density distribution of whole-brain resting-state functional connectivity. gFCD is thought to reflect the brain’s ability to process information (Qin et al. 2015) and can be considered a biomarker for quantitative state changes in glucose metabolism (Thompson et al. 2016). gFCD has been used to investigate brain functional connectivity alterations in several mental disorders (Cohen et al. 2018; Huang et al. 2017), including schizophrenia with AVH (Zhuo et al. 2019). Hence, in the present study we adopted gFCD to investigate the common and distinct functional brain patterns in Hi-AVH with and without insight.

## Methods

### Samples

This study was approved by the ethics committee of Wenzhou Seventh People’s Hospital. All subjects were provided with detailed information on study process and purpose, and all subjects gave their informed consent. A total of 50 Hi-AVH and 50 AVH-free controls were enrolled to participate in the study by advertising in the community from July 2017 to December 2018. Hi-AVH inclusion criteria were as follows: 1) have AVH satisfying the proposed criteria by Johns “Did you at any time hear voices saying quite a few words or sentences when there was no one around that might account for it?”; 2) no other psychotic symptoms as determined by structured clinical interviews using the DSM-IV Axis I Disorders-Patient Edition (SCID-I/P) conducted by two senior psychiatrists with more than 10 years of experience; 3) IQ >80. The exclusion criteria were as follows: 1) other psychotic or affective disorders, mental retardation, alcohol dependence, drug dependence, organic brain lesions, or physical and neurological diseases; 2) history of unconsciousness for more than 5 min caused by any reason; 3) contraindications for MRI examination; 4) claustrophobia; 5) IQ < 80. All subjects were right-handed. Controls were distinguished by a professional psychiatrist using the SCID non-patient version.

### Assessment of AVH severity

In this study, AVH severity was assessed using the auditory hallucinations rating scale (AHRS) (Hoffman et al. 2000). All subjects were assessed by a trained psychiatrist for affective and psychotic DSM-IV axis I pathologies using the Comprehensive Assessment of Symptoms and History (CASH) (Larøi et al. 2004). Global functioning was estimated using the Global Assessment of Functioning (GAF) scale (Andreasen et al. 1992), on which normal, healthy adults typically score above 90. To assess axis II pathologies, SCID-II was added to the psychiatric screening 6 months after the start of the study (First et al. 1995). The presence of psychiatric disorders in family members of the participants were quantified using the Family Interview for Genetic Studies (Takahashi et al. 2005). Urine samples were obtained to screen for drugs of abuse (including cannabis, amphetamine, cocaine, methadone, and heroin), the presence of which were grounds for exclusion from the study. Individuals with psychotic DSM-IV axis I and axis II pathologies or a family history of psychiatric disorders were also excluded. The Insight and Treatment Attitudes Questionnaire (ITAQ) was used to distinguish “with insight” vs. “without insight”. An ITAQ score of 22 was defined as having full insight, while a score of 0 was defined as having no insight. In this study, to maximize the distinction between groups, we selected only those patients with full or no insight, and discarded individuals with partial insight.

### MRI data acquisition

Functional magnetic resonance imaging (fMRI) was performed on a 3 T GE Discovery MR750 scanner (General Electric, Milwaukee, WI, USA) equipped with an eight-channel phased-array head coil. The participants were instructed to lie down in a supine position and to rest without falling asleep during the scan. Whole-brain resting-state fMRI data depicting blood oxygen level-dependent signals were obtained using a gradient-echo echo-planar imaging sequence with the following parameters: repetition time (TR) = 2000 msec; echo time (TE) = 45 msec; slices = 32; slice thickness = 4 mm; gap = 0.5 mm; field of view (FOV) = 220 × 220; matrix size = 64 × 64; and flip angle (FA) = 90°. All scans were acquired by parallel imaging using the sensitivity encoding (SENSE) technique with a SENSE factor of 2. Structural images were obtained with a high-resolution 3D Turbo-Fast Echo T1WI sequence with the following parameters: 188 slices, TR/TE = 8.2/3.2, slice thickness = 1 mm, no gap, FA = 12°, matrix size = 256 × 256, FOV = 256 × 256.

### Data preprocessing

SPM8 software was used to process the resting-state fMRI scans (http://www.fil.ion.ucl.ac.uk/spm). To allow for imaging unit stabilization and subject familiarization, the first 10 volumes of each scan were discarded. The remaining volumes were corrected for slice-timing and motion artifacts. Head translation movement for all participants was less than 2 mm, and rotation was less than 2°. Covariates, including head motion, white matter signal, and cerebrospinal fluid signal, were regressed out from the time series of every voxel. The Friston 24-parameter model was used to regress out head motion effects. Data were regressed out if the framewise displacement of a specific volume was >0.5. The datasets were filtered with band pass frequencies ranging from 0.01 to 0.08 Hz. Individual structural images were co-registered to the mean functional image, and the transformed structural images were co-registered to Montreal Neurological Institute (MNI) space using a linear registration. Motion-corrected functional volumes were spatially normalized to MNI space using parameters estimated during the linear co-registration. Finally, the functional images were re-sampled into 3 mm cubic voxels for further analysis.

### Calculation of gFCD

The functional connectivity of each voxel was calculated using an in-house Linux script as previously reported; briefly, Pearson’s linear correlation was applied with a correlation coefficient threshold of *R* > 0.6 (Tomasi et al., 2010; Zou et al., 2016). The gFCD calculations were limited to those voxels within the cerebral gray matter mask, and the gFCD at any given voxel (×0) was calculated as the total number of functional connections, denoted as k (×0), between ×0 and all other voxels using a growth algorithm, which was repeated for all of the ×0 voxels. Next, gFCD was divided by the mean value of each voxel in the brain to increase the normality of the distribution. The FCD maps were spatially smoothed with a 6 × 6 × 6 mm^3^ Gaussian kernel to minimize differences in brain anatomy between subjects.

### Statistical analysis

Group differences in gFCD among the three groups were tested using a voxel-wise one-way analysis of covariance (ANCOVA) with age, gender, and education level as covariates, followed by post hoc intergroup comparisons. The post hoc intergroup comparisons were conducted within a mask showing gFCD differences from the ANCOVA analysis, and T value was used to aid in contrast indicators (Tomasi et al., et al., 2010). Multiple comparisons were corrected using the family wise error (FWE) method with a significance threshold of P<0.05.

To investigate the relationship between gFCD and total AHRS scores, a voxel-wise multiple regression analysis was conducted in the Hi-AVH group within regions showing significant gFCD differences compared with the other two groups. Gender, age, and education level were considered as nuisance covariates. Multiple comparisons were corrected using the FWE method with a significance threshold of P<0.05. To investigate the relationship between gFCD and ITAQ scores, a voxel-wise multiple regression analysis was conducted in the Hi-AVH with insight group within regions showing significant gFCD differences compared with the other two groups. Gender, age, GAF score, and education level were considered as nuisance covariates. Multiple comparisons were corrected using the FWE method with a significance threshold of P<0.05.

Differences in the demographic measurements among these three groups were examined using Chi-square test (gender) or one-way ANOVA (age and education level). Differences in the social demographic information between the two Hi-AVH groups were tested using a two-sample t-test.

## Results

### Demographic and clinical characteristics

Sixteen Hi-AVH with insight, 17 Hi-AVH without insight, and 20 healthy controls were enrolled in the analysis. These three groups were matched in terms of gender (*X*^*2*^ = 0.198, *P* = 0.911), age (F = 0.209, *P* = 0.903), and education level (F = 0.452, *P* = 0.520). There were no significant differences in AVH severity (t = 2.175, *P* = 0.423) or duration (t = 1.010, *P* = 0.211) between the Hi-AVH groups with and without insight. There was, however, a significant difference in GAF score between groups (*P* < 0.05; Table 1).

**Table 1:** Social-demographic information

Characteristics	Hi-AVH with insight (*n* = 13)	Hi-AVH without insight (*n* = 15)	AVH-free controls (n = 15)	*P*
Age (years)	22.5 (2.0)	23.7 (2.6)	22.3 (1.5)	0.903
Gender (female/male)	6/7	8/7	8/7	0.911
Duration of AVH (days)	125.5 (40.5)	130.0 (51.0)	N/A	
Education level (years)	10.3 (2.5)	12.9 (2.0)	13.0 (3.1)	0.520
Auditory Hallucination Rating Scale total score	20.7 (3.5)	22.4(2.8)	N/A	
GAF score	82.2 (10.6)	75.4 (12.5)	100.0 (0.0)	*p* < 0.001

Values are expressed as mean (s.d.)

### gFCD differences

**Fig. 1:**
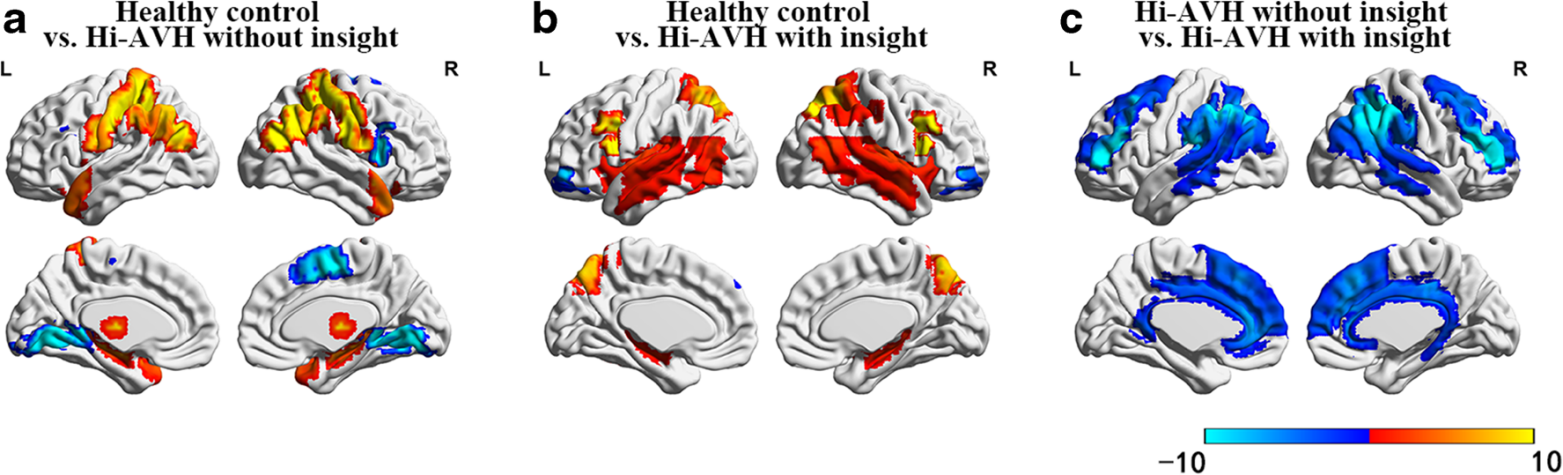
gFCD differences between groups, with T-values indicated by color. Figure 1a: Healthy controls vs. Hi-AVH without insight; Fig. 1b: Healthy controls vs. Hi-AVH with insight; Fig. 1c: Hi-AVH without insight vs. Hi-AVH with insight

Compared to AVH-free controls, the Hi-AVH group without insight showed higher gFCD located mainly in the postcentral gyrus, superior parietal lobule, superior temporal gyrus, and temporal pole, and lower gFCD located in the superior frontal gyrus and lingual gyrus (Fig. 1a). Compared to AVH-free controls, the Hi-AVH group with insight showed higher gFCD in the inferior frontal gyrus, superior parietal lobule, superior temporal gyrus, and angular gyrus, and lower gFCD in frontal pole (Fig. 1b). The most common gFCD alterations between Hi-AVH groups and controls involved the superior parietal lobule and superior temporal gyrus. We therefore considered these alterations to be common brain functional pathological features of AVH. Differences in gFCD between the two Hi-AVH groups were located in the postcentral gyrus, temporal pole, superior frontal gyrus, and lingual gyrus. We therefore defined these regions as insight-related (FWE corrected at voxel level, P<0.05).

Compared to Hi-AVH with insight, Hi-AVH without insight had lower gFCD in the frontal lobe oculomotor area, dorsolateral prefrontal cortex, supramarginal gyrus, primary auditory cortex, sensorimotor cortex, ventral anterior, and posterior cingulate (Fig. 1c). We defined these alterations as distinctive features of Hi-AVH without insight.

### Correlation analysis

We did not observe any significant correlation between gFCD and AVH severity in either Hi-AVH group.

## Discussion

In this pilot study, we aimed to detect the common and distinct intrinsic functional features in Hi-AVH with and without insight. We observed that gFCD alterations in the Hi-AVH group without insight were more severe than in those with insight.

Brain regions that were altered in both Hi-AVH groups were ones involved in perception, memory, language, and central executive control. These findings support the “Resting-State Hypothesis of AVH III: Reduced Rest-external Stimulus Interaction in the Auditory Cortex” (Northoff G, et al., 2014; Branislava Ćurčić-Blake et al. 2017; Ben et al. 2016; Bohlken et al. 2017; Bose et al. 2017; Hugdahl and Sommer 2018; Kubera et al. 2019; NIMH 1992). This hypothesis postulates that abnormal strong rest-rest interactions in auditory cortex are perceived as externally, rather than internally, derived voices.

Our findings also support the hypothesis that abnormal reciprocal action of the perception, memory, language, and central executive networks can contribute to AVH (Alderson-Day et al. 2015; Hugdahl 2015; Ćurčić-Blake et al. 2017; Hugdahl 2017; Alderson-Day et al. 2016). Compared to the Hi-AVH group with insight, the Hi-AVH group without insight showed greater disturbance within these networks, and also within components of mood regulation circuitry. Previous studies have reported that functional alterations in these circuits are not only related to AVH but also to insight (Webb et al. 2016; Korovkin et al. 2018; Hill and Kemp 2018; Belvederi Murri et al. 2016; Tik et al. 2018; Sapara et al. 2016).

We did not observe any significant relationship between ARHS severity and alterations in gFCD, although such a relationship has been reported by others (Lefort-Besnard et al. 2018; Torres et al. 2016; Torres et al. 2016; Wei et al. 2016). It is possible that brain alterations as measured by gFCD may only offer a qualitative index, not a quantitative one, of AVH. Further studies are needed to clarify this point.

There are several limitations to this pilot study. First, the small sample size limits the generalizability of our findings. Second, since it was only a cross-sectional study, prospective longitudinal studies will be necessary to more fully describe the relationship between functional brain alterations and clinical symptoms and to explore early intervention strategies.

## Conclusion

To the best of our knowledge, this is the first study focusing on alterations in functional connectivity density in Hi-AVH with and without insight. The salient findings of this pilot study were that there are indeed common and distinct brain functional alterations related to insight. Moreover, these findings support the hypothesis that abnormal reciprocal action of perception, memory, language, and attentional-control circuits contributes to the pathological features of AVH. Although some limitations existed, our pilot study nevertheless provided important clues to guide further studies.
